# Tracking mood fluctuations with functional network patterns

**DOI:** 10.1093/scan/nsy107

**Published:** 2018-11-27

**Authors:** Nykan Mirchi, Richard F Betzel, Boris C Bernhardt, Alain Dagher, Bratislav Mišić

**Affiliations:** 1 McConnell Brain Imaging Centre, Montréal Neurological Institute, McGill University, Montréal, QC, Canada; 2 Department of Psychological, and Brain Sciences, Indiana University, Bloomington, IN, USA

**Keywords:** connectome, mood, deep phenotyping, fMRI, connectivity

## Abstract

Subjective mood is a psychophysiological property that depends on complex interactions among the central and peripheral nervous systems. How network interactions in the brain drive temporal fluctuations in mood is unknown. Here we investigate how functional network configuration relates to mood profiles in a single individual over the course of 1 year. Using data from the ‘MyConnectome Project’, we construct a comprehensive mapping between resting-state functional connectivity (FC) patterns and subjective mood scales using an associative multivariate technique (partial least squares). We report three principal findings. First, FC patterns reliably tracked daily fluctuations in mood. Second, positive mood was marked by an integrated architecture, with prominent interactions between canonical resting-state networks. Finally, one of the top-ranked nodes in mood-related network reconfiguration was the subgenual anterior cingulate cortex, an area commonly associated with mood regulation and dysregulation. Altogether, these results showcase the utility of highly sampled individual-focused data sets for affective neuroscience.

## Introduction

The brain’s white matter architecture promotes coherent signaling among neuronal populations, enabling perception, cognition and action (Fries, 2015; Avena-Koenigsberger *et al.*, 2018). Non-invasive measurements of electromagnetic and hemodynamic neural activity permit comprehensive mapping of statistical interactions among distributed areas, termed functional connectivity (FC). In the absence of overt sensory stimulation or task demands, brain areas spontaneously synchronize to form networks with specific functional characteristics, termed intrinsic connectivity networks or resting-state networks (RSNs; Damoiseaux *et al.*, 2006; Power *et al.*, 2011; Yeo *et al.*, 2011; Cole *et al.*, 2014). Recent reports demonstrate that resting FC patterns are heritable (Glahn *et al.*, 2010; Ge *et al.*, 2017) and can even be used to identify individuals, much like a fingerprint (Miranda-Dominguez *et al.*, 2014; Finn *et al.*, 2015). A significant body of work has emerged linking resting FC patterns to individual differences in cognitive performance (Smith *et al.*, 2015; Mišić and Sporns, 2016; Rosenberg *et al.*, 2016).

Much like cognition, subjective affect (here referred to as ‘mood’) also arises from a complex set of network interactions. A fundamental psychophysiological property, mood depends on distributed signaling throughout the central and peripheral nervous systems, involving interoception, emotional state and memory (Critchley, 2005; Kragel *et al.*, 2018). These complex interactions are conditioned by the underlying anatomical connectivity (Joyce and Barbas, 2018). Altered communication throughout this neural circuit is thought to be the origin of mood disorders such as major depressive disorder (MDD; Berman *et al.*, 2014; Mulders *et al.*, 2015). In the cerebral cortex, mood regulation and dysregulation is associated with several key areas, particularly the subgenual portion of the anterior cingulate cortex (sgACC; Brodmann area 25; Mayberg *et al.*, 1999; Price and Drevets, 2012). The circuit embedding of these areas means that pathological perturbations, such as MDD, manifest at the level of large-scale RSNs, including the salience and default mode networks (Mulders *et al.*, 2015). Indeed, areas such as sgACC and their connected neighbors have been proposed as targets for deep brain stimulation to treat MDD (Mayberg *et al.*, 2005; Fox *et al.*, 2014; but see also Holtzheimer *et al.*, 2017).

Systematically relating mood to distributed FC patterns poses a unique challenge. By definition, mood is dynamic, fluctuating over minutes, hours and days. To statistically relate fluctuations in mood with FC patterns, repeated intra-individual sampling is necessary. A recent landmark data set makes such an investigation possible. In the `MyConnectome Project', a single individual (a healthy 45-year-old male) was phenotyped over the course of several months (Laumann *et al.*, 2015; Poldrack *et al.*, 2015). Weekly functional magnetic resonance imaging (fMRI) scans were accompanied by self-reported measures of affect [expanded Positive and Negative Affect Schedule (PANAS-X); Watson and Clark, 1999] as well as a comprehensive battery of physiological measurements. To date, two studies have investigated the relation between FC and mood in this context. Shine *et al.* (2016b) examined the propensity for regions to switch allegiance among large-scale networks within recoding sessions and related this property to self-reported attention. Betzel *et al.* (2017) investigated a similar within-session flexibility measure in relation to subjective mood ratings and found that greater flexibility was associated with positive mood scales. How daily fluctuations in connectivity track mood and how these connectivity patterns are anatomically organized, remains unknown.

In the present report we apply an associative multivariate technique [partial least squares (PLS); McIntosh and Lobaugh, 2004; McIntosh and Mišić, 2013] to the ‘MyConnectome’ data set to isolate patterns of functional connections and mood profiles that fluctuate together across time. We then characterize the topological organization of mood-related connectivity patterns and identify their epicenters. Finally, we assess the signature of multiple cognitive and affective systems and how their relative balance is related to positive and negative mood.

## Results

Subjective mood ratings were organized into 13 distinct positive and negative scales as proscribed by Watson and Clark (1999): negative affect, positive affect, fear, hostility, guilt, sadness, joviality, self-assurance, attentiveness, shyness, fatigue, serenity and surprise. fMRI data were pre-processed and parceled into 630 cortical and subcortical regions of interest. FC was defined as a zero-lag linear Pearson correlation between regional blood oxygenation level dependent (BOLD) signal time series. Only sessions that had complete fMRI and mood scales were analyzed (total, 73). The upper triangle of FC correlation matrices was submitted to PLS analysis. A form of reduced-rank linear regression, PLS seeks to maximize the relation between individual functional connections and mood scales in a single multivariate pattern (McIntosh and Mišić, 2013).

### Network configuration tracks mood fluctuations

**Fig. 1:**
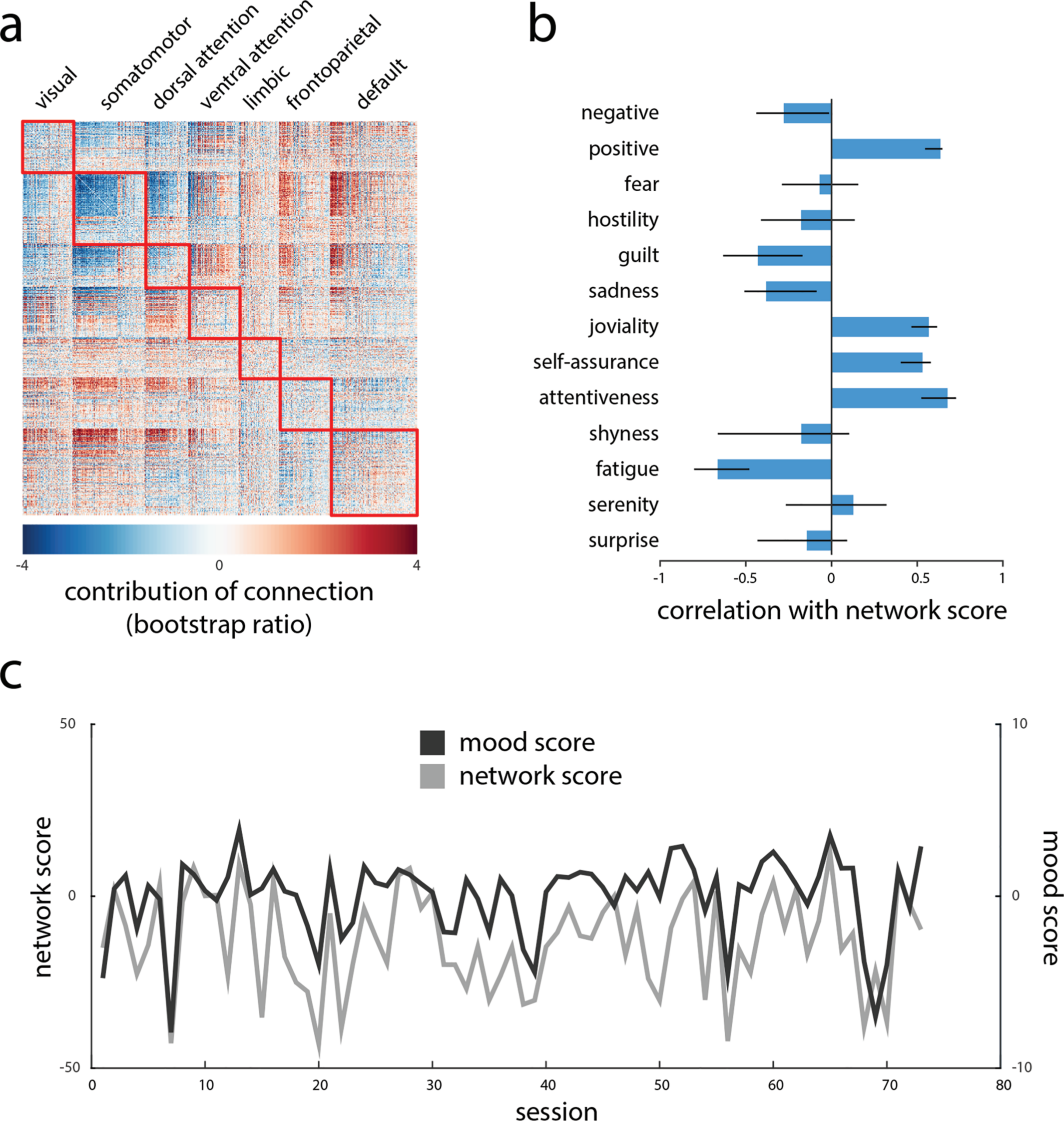
Connectivity patterns track mood patterns. Multivariate PLS analysis was used to isolate patterns of functional connections and PANAS-X mood scales that maximally covary with each other. **(A)** Functional connections that correlate with positive mood (red) and negative mood (blue). Connections are weighted by bootstrap ratios (singular vector weight divided by its bootstrap-estimated standard error). **(B)** Correlations (i.e. loadings) of PANAS-X mood scales with the functional network pattern. Error bars represent bootstrap-estimated 95% CIs. **(C)** Network and behavioral responses or scores are estimated for each individual session by projecting the session data onto the singular vectors. The scores index the extent to which the participant expressed functional patterns and mood patterns shown in (A) and (B).

PLS analysis revealed a statistically significant association between FC and mood across 73 sessions (permuted *P ≈* 0) accounting for 52% of the covariance between connectivity and mood. Figure 1 shows (i) functional connections and (ii) mood scales that contribute most to this pattern. Functional connections are weighted by bootstrap ratios, a measure of reliability (see ‘Materials and methods’ for details).

Elements (connections or mood scales) weighted with the same sign covary positively, while those with opposite signs covary negatively. In other words, connections with positive weights (red) are associated with greater positive mood (e.g. positive, joviality, self-assurance and attentiveness), while connections with negative weights (blue) are associated with greater negative mood (e.g. negative, guilt, sadness and fatigue). Nodes are arranged by their membership in RSNs derived by Yeo *et al.* (2011; Figure 1A).

To further illustrate the relation between connectivity and mood, Figure 1C shows fluctuations in individual session scores for both patterns. The scalar scores are calculated by projecting individual session data onto the PLS-derived pattern. They reflect the extent to which a given statistical pattern (e.g. functional network or mood profile) is expressed in a given session. The close correspondence between the network and mood scores suggests that when the participant exhibited network configurations similar to Figure 1A (gray), he was more likely to exhibit the mood pattern in Figure 1B (black; *r* = 0*.*68).

### Regional contributions to mood-related connectivity

**Fig. 2:**
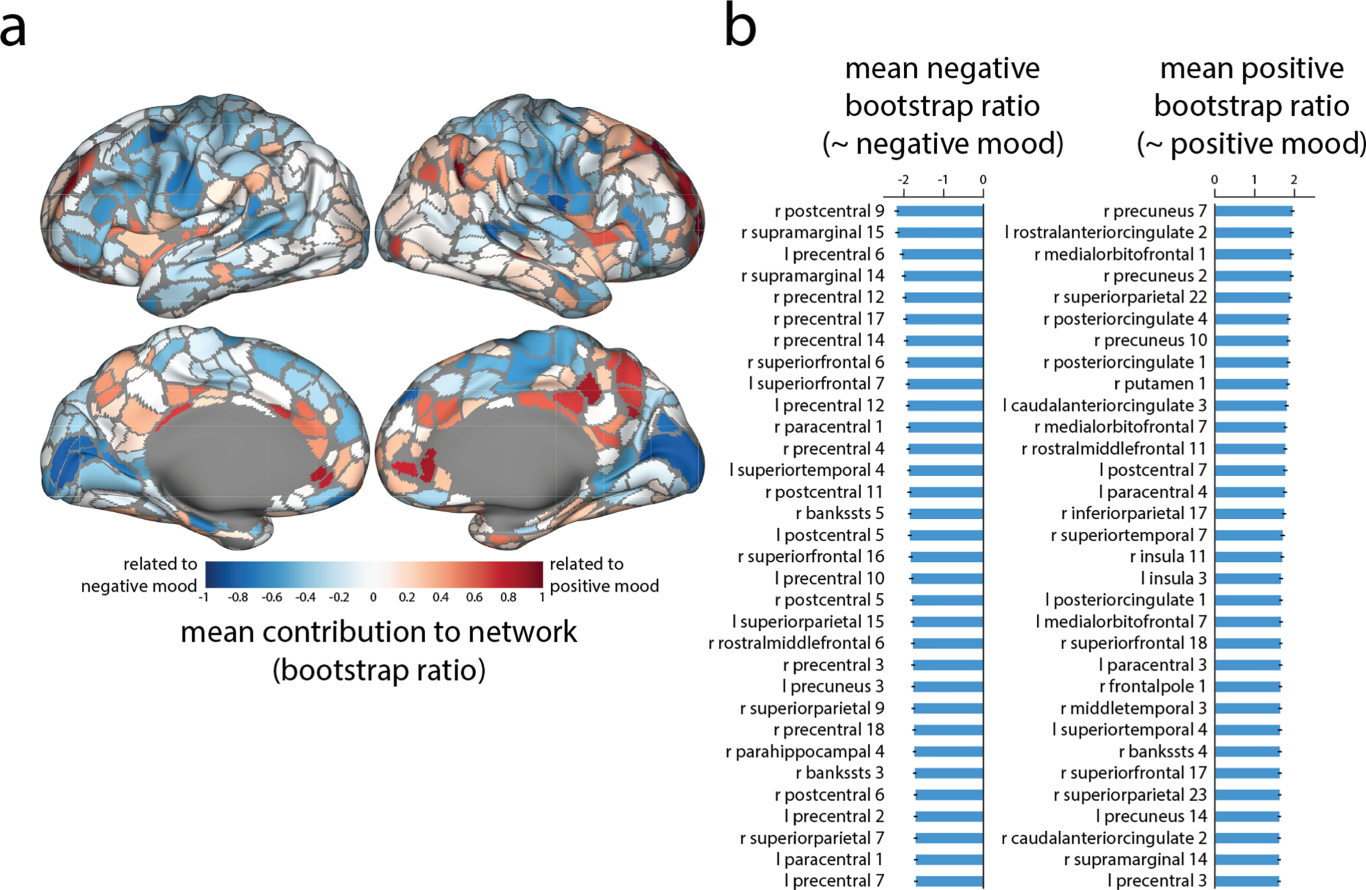
Regional contributions to mood-related network patterns. **(A)** Mean bootstrap ratios of all connections at each region (estimated from Figure 1A). Positive values are associated with more positive mood states, while negative values are associated with negative mood states. **(B)** Top 5% of nodes with the greatest mean negative and positive bootstrap ratio. CIs indicate standard errors.

We next investigated the contribution of individual brain regions to the mood-related functional network pattern. Figure 2A shows the mean bootstrap ratio (i.e. network contribution) of all functional connections that a given region participates in. Areas with predominantly positive values, such as precuneus, anterior cingulate cortex and medial orbitofrontal cortex, are more prominent in the global network during positive mood. Conversely, areas with negative values, such as paracentral cortex, lateral prefrontal cortex and visual cortex, feature prominently during negative mood. Figure 2B confirms this intuition, showing the top 5% nodes with the greatest mean negative, positive and absolute bootstrap ratio. We note that the positive mood-related pattern closely resembles the default mode network (Greicius *et al.*, 2003; Fox *et al.*, 2005).

**Fig. 3:**
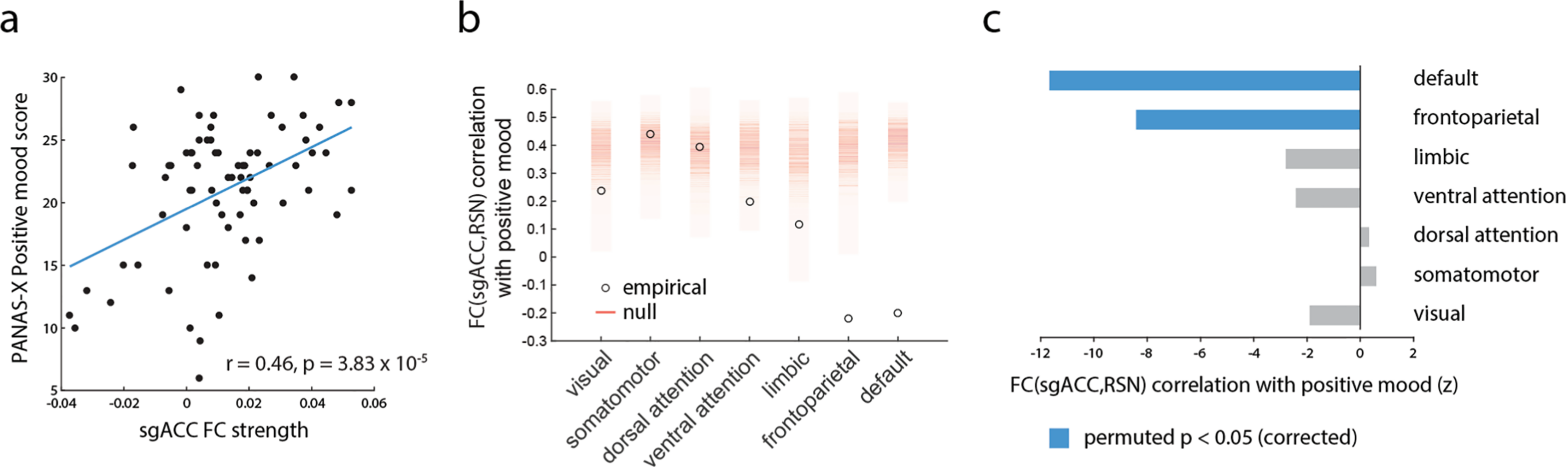
Network embedding of sgACC is associated with mood fluctuations. Based on regional rankings in Figure 2 and prior literature, we investigate the role of sgACC. **(A)** sgACC FC strength (average correlation to other brain areas) correlates with PANAS-X positive mood score across sessions. **(B)** Correlations between positive mood on one hand and FC between sgACC and canonical RSNs on the other. Empirical correlations are displayed as circles; ‘null’ correlations, obtained by permuting RSN labels, are shown as a histogram (red). FC between sgACC and default mode and frontoparietal networks correlates with positive mood significantly less than expected by chance. **(C)** Empirical correlations between FC (sgACC,RSN) and positive mood are shown as *z*-scores relative to a label-permuting null distribution.

Given the high ranking of sgACC in Figure 2B, and its prominent role in mood disorders (Mayberg *et al.*, 2005; Price and Drevets, 2012; Mulders *et al.*, 2015), we next examine its connectivity fingerprint. Figure 3A shows a positive correlation between sgACC FC strength (FC with other nodes) and the PANAS-X positive mood score (*r* = 0*.*46; *P* = 3*.*83 × 10^−5^), indicating that overall integration of sgACC was associated with better mood. Note that this relationship is fully expected given the results presented in Figure 2B and cannot be interpreted as an independent discovery (Vul *et al.*, 2009).

Finally, we ask how FC between sgACC and specific large-scale systems relates to mood. Figure 3B shows how mean sgACC-RSN FC correlates with positive mood (circles). While connectivity with some RSNs was associated with greater positive mood, for others the relationship was reversed, suggesting that sgACC integration did not uniformly predict better mood. To assess the statistical significance of this connectivity fingerprint, we also compute correlations between sgACC-RSN connectivity and positive mood under a null model where RSN labels are randomly permuted for all nodes (Figure 3B; red density). When empirical correlations are expressed as z-scores relative to the null distribution, only the negative correlations observed between sgACC and the default mode and frontoparietal networks are statistically significant (Figure 3C; permuted *P ≈* 0 for both). Altogether, these results demonstrate a complex reconfiguration of sgACC connectivity; positive mood is associated not only with a diffuse, non-specific integration of sgACC with other brain regions but also with a specific decorrelation of sgACC from the default mode and frontoparietal networks.

### Positive mood is associated with network integration

Rearranging brain areas by their membership in RSNs (e.g. Figure 1A) suggests that modular structure may potentially be associated with mood. To further investigate whether network integration shapes the relationship between connectivity patterns and mood, we partitioned the network into RSNs (Yeo *et al.*, 2011) and stratified functional connections into those connecting areas in the same intrinsic network or different networks.

**Fig. 4:**
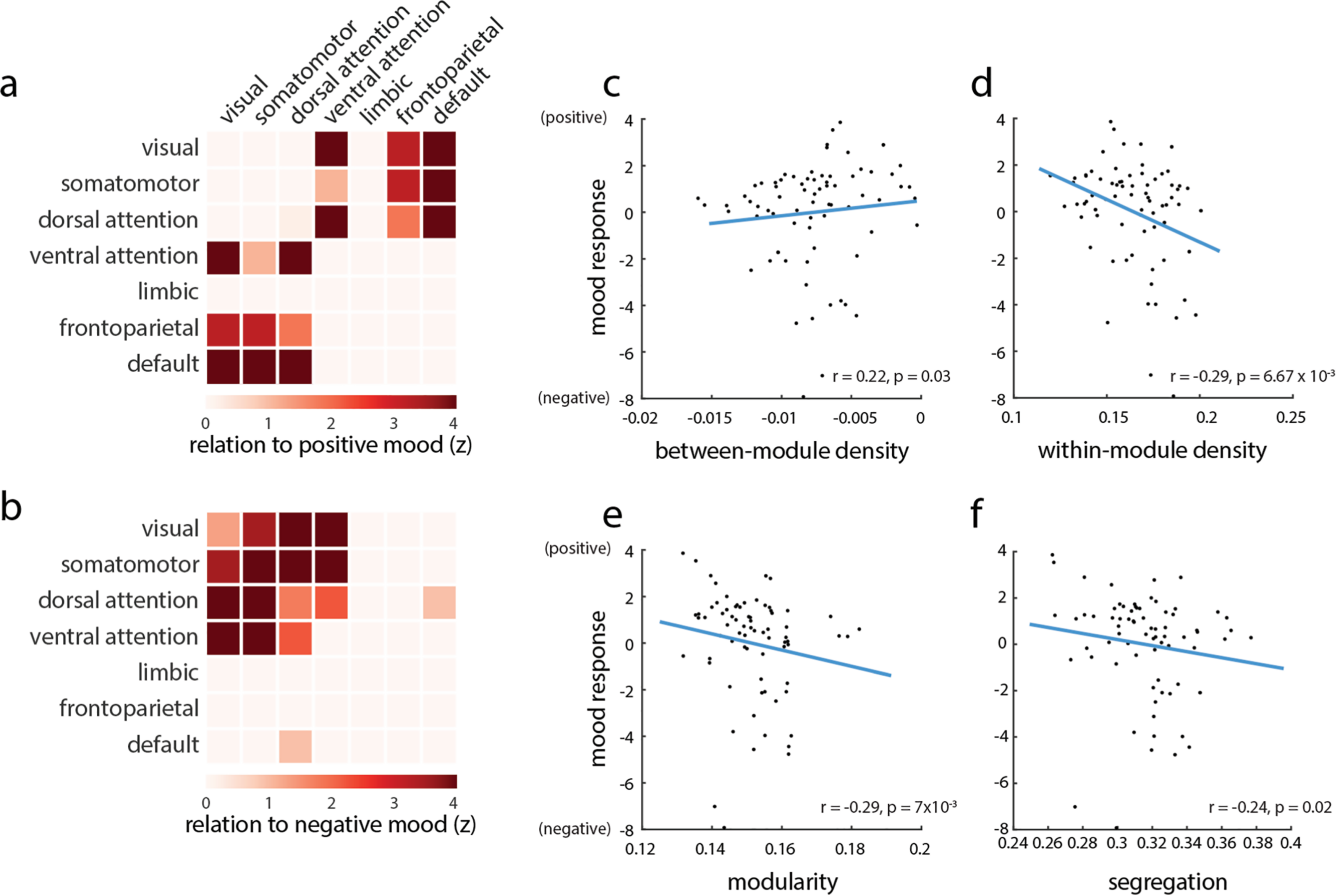
Intrinsic network contributions to mood fluctuations. Bootstrap ratio-weighted functional networks are partitioned according to the RSN assignments reported by Yeo *et al.* (2011). **(A)** Mean positive bootstrap ratios within and between RSNs, expressed as a *z*-score relative to a label-permuting null model. High values indicate greater than expected network connectivity during positive mood states. **(B)** The same procedure is performed but focusing on connections related to negative mood. High values indicate greater than expected network connectivity during negative mood states. (**C** and **F**) Correlating network segregation and integration with behavioral responses (high values are equal to positive mood; low values are equal to negative mood) in individual sessions. Network integration (between-module connectivity) is associated with positive mood, while network segregation (measured by within-module connectivity, modularity and segregation) is associated with negative mood.

To assess whether RSN–RSN interactions were statistically significant, we used a label-permuting null model. We randomly permuted RSN labels for each node and recalculated the mean bootstrap ratio for connections within and between RSNs (10 000 repetitions; Mišić *et al.*, 2015; 2016). Figure 4A and B show the mean connection bootstrap ratio for positive mood-related and negative mood-related weights, respectively. The weights are expressed as z-scores relative to the label-permuting null distribution. The resulting figure suggests that positive mood is generally associated with between-RSN connectivity, while negative mood is associated with within-RSN connectivity, consistent with Figure 1A.

We next investigated the possibility that network integration contributes to positive mood, while network segregation contributes to negative mood. For each experimental session, we correlated the mean value of within- and between-module connections with the session-specific score on the PLS-derived behavioral pattern, corresponding to positive mood. Greater between-module connectivity was associated with positive mood (Spearman *r* = 0*.*22; *P* = 0*.*03; Figure 4C), while greater within-module connectivity was associated with negative mood (Spearman *r* = 0*.*29; *P* = 6*.*67 × 10^−3^; Figure 4D). As two complementary measures of network segregation, we computed the modularity and system segregation of FC networks in individual sessions (see ‘Materials and methods’; Newman and Girvan, 2004; Chan *et al.*, 2014). Modularity—the tendency for nodes to connect with other nodes in the same RSN—was associated with negative mood (Figure 4E; Spearman *r* = 0*.*29; *P* = 0*.*007). Similarly, system segregation—the normalized difference of within- and between-RSN connectivity—was also associated with negative mood (Figure 4F; Spearman *r* = *−*0*.*22; *P* = 0*.*02).

It is possible that these effects are not module-dependent but rather trivially driven by non-specific fluctuations in network density. To test this hypothesis, we permuted module labels but kept the permuted labels the same across all sessions. We then recalculated the correlations between mood and within-/between-module density, repeating the procedure 10 000 times. The resulting distribution of correlation coefficients embodies the null hypothesis that density fluctuations drive the observed correlations, as opposed to module assignments. For all four measures (within-module density, between-module density, modularity and segregation), the empirical correlation was significantly greater than expected under the null model (permuted *P ≈* 0 for all four measures). Altogether, these results indicate that positive mood was broadly associated with greater network integration (higher between-network connectivity) and lower network segregation (higher within-network connectivity).

### Stability of functional and mood patterns

Relating two sets of variables when the number of variables is larger relative to the number of observations poses a risk of overfitting and may result in a model that does not generalize to future observations. Although pattern significance and reliability are assessed by permutation and bootstrapping, respectively, the correlation between brain and behavioral patterns may be optimistic because it is maximized by the analysis.

Here we assess two properties related to stability. First, we assess the stability of the connectivity and behavioral patterns via split-half resampling (Kovacevic *et al.*, 2013; see ‘Materials and methods’ for more details). Briefly, the sample is split into halves and the decomposition is performed on each half separately. Data from one split are then projected onto the left and right singular vectors (corresponding to the connectivity and mood patterns) calculated from the other split. Pattern stability is quantified as the mean correlation between left/right singular vectors directly calculated from one split and left/right singular vectors calculated by projecting the other split onto the corresponding right/left singular vector. The mean correlation among projected network patterns was *r* = 0*.*26 [95% confidence interval (CI): 0.11 0.37], while the correlation among mood patterns was *r* = 0*.*91 (95% CI: 0.80 0.94), suggesting that both sets of patterns were stable across splits.

We next assess the out-of-sample correlation between connectivity and mood scores. Following the method described by Rahim *et al.* (2017), we reran the analysis with 100 randomized train and test splits, where test sets represent 25% of the sample. Test data were projected on the PLS models constructed on the training set. Predicted mood and connectivity scores were then correlated; the resulting mean out-of-sample mood–connectivity correlation was *r* = 0*.*34. The correlation was statistically significant against a permutation test (permuted *P ≈* 0).

### Effects of fasting and lifestyle variables

To facilitate blood tests, the participant did not consume food or caffeine on Tuesday sessions, resulting in a roughly equal split between fasting and non-fasting sessions (39 *vs* 34). Two previous reports indicated that fasting could alter functional network architecture (Poldrack *et al.*, 2015; Betzel *et al.*, 2017), suggesting that the present results could be influenced by fasting. To assess this possibility, we directly compared PLS-derived scores on fasting and non-fasting days. Two-sample *t*-tests confirmed significantly different functional network expression on fasting and non-fasting days [*t*(71) = 4*.*00; *P* = 1*.*5 × 10^−4^]. There was, however, no evidence of significant differences with respect to mood expression [*t*(71) = 1*.*00; *P* = 0*.*32]. Importantly, PLS-estimated connectivity and mood pattern scores were correlated on both fasting (*r* = 0*.*67; *P* = 2*.*75 × 10^−5^) and non-fasting (*r* = 0*.*74; *P* = 7*.*13 × 10^−7^) days, suggesting that the relation between connectivity and mood was present independent of whether the participant fasted or not. Fisher’s test indicated that the two correlation coefficients were not significantly different (*z* = 0*.*51; *P* = 0*.*61). Altogether, this suggests that fasting influenced functional network patterns but did not influence mood patterns or the pairing between connectivity and mood patterns.

It is possible that fluctuations in mood and FC depend on a variety of other health and lifestyle variables. To investigate this possibility, we correlated mood scores and network scores separately with variables recorded on evenings before and evenings of the MRI scans. Variables include both subjective and objective measures: same and previous evening subjective measures (alcohol consumption, gut health, psoriasis severity, stress and time spent outdoors), weather (precipitation and lowest and highest temperature) and sleep quality [total sleep time, time in light sleep, deep sleep and rapid eye movement (REM) sleep; Poldrack *et al.*, 2015]. The linear regression analysis revealed no statistically significant associations [*P >* 0*.*05 for all comparisons; false discovery rate (FDR) corrected], suggesting that fluctuations in mood and connectivity are unlikely to be explained by these exogenous factors.

## Discussion

In the present report we investigated how FC and mood covary in a single individual over the course of 1 year. We find that fluctuations in mood are reliably tracked by functional network organization. Positive mood was associated with greater integration between canonical intrinsic networks. The sgACC was a highly-ranked node in the isolated network patterns, consistent with its role in rumination and clinical depression.

### Functional network organization and mood

The present results add further evidence that subjective mood is an integrated network property, arising from complex communication patterns throughout the cerebral cortex (Critchley, 2005; Shine *et al.*, 2016b; Betzel *et al.*, 2017). Specifically, mood is thought to arise from a set of polysensory circuits linking interoception, emotion and memory (Critchley, 2005). We find evidence of organized FC network patterns that systematically reconfigure in parallel with mood changes. Positive mood was associated with increased coherence among anterior medial (gACC) and posterior medial (posterior cingulate) structures (Figure 1). More broadly, we find that increased coherence between intrinsic networks (integration) was associated with positive mood, while increased coherence within intrinsic networks (segregation) was associated with negative mood (Figure 4). It is noteworthy that affinity toward integrated states or configurations is also associated with better cognitive performance (Shine *et al.*, 2016a). The present results complement a previous investigation using the ‘MyConnectome’ data set. Betzel *et al.* (2017) reported that moment-to-moment network flexibility was associated with positive mood indices. Our results show that a relationship between network structure and mood can be observed over longer temporal scales as well. Importantly, we find that positive mood is not necessarily associated with random reconfigurations but specifically with a tendency toward less segregated/more integrated states.

These results contribute to a growing literature linking functional network interactions with cognition and affect (Medaglia *et al.*, 2015; Mišić and Sporns, 2016). A number of recent studies have demonstrated that connectivity patterns predict multiple aspects of cognitive performance including fluid intelligence (Smith *et al.*, 2015), cognitive control (Cole *et al.*, 2012), attention (Rosenberg *et al.*, 2016), working memory (Greicius *et al.*, 2003) and learning (Bassett *et al.*, 2015). How functional connections are arranged and rearranged to represent and interact with the external environment remains a key question (Mišić *et al.*, 2014; Zalesky *et al.*, 2014; Shine and Poldrack, 2017).

While ostensibly healthy, the participant’s network patterns observed in relation to positive and negative moods mirror those that have been reported in clinical MDD. For example, multiple studies have reported increased FC between sgACC and the default mode network in MDD (Zhou *et al.*, 2010; Davey *et al.*, 2012; de Kwaasteniet *et al.*, 2013), a pattern we observe in relation to decreased positive mood (Figure 3). The increased integration of sgACC effectively changes the configuration of the default mode network in MDD (Greicius *et al.*, 2007). Moreover, treatments such as repetitive transcranial stimulation reduce sgACC default connectivity (Liston *et al.*, 2014; Salomons *et al.*, 2014), consistent with the positive mood-related patterns we observe. Altogether, these potential overlaps suggest that healthy daily fluctuations in mood may be a microcosm of clinical mood disorders.

More generally, the reconfiguration of sgACC connectivity demonstrates how systems-level effects can obscure complex connection ‘fingerprints’ of individual nodes. In the present analysis we find that general default mode connectivity is associated with positive mood (Figure 2); likewise, mean sgACC connectivity (Figure 3A) is also associated with positive mood. However, their mutual connectivity is anticorrelated with positive mood (Figure 3B and C). In other words, the integration of sgACC and other default regions with the rest of the brain is associated with positive mood, but their mutual connectivity is associated with negative mood. How these complex reconfiguration patterns map onto the heterogeneous connection profile of sgACC remains an exciting open question (Palomero-Gallagher *et al.*, 2018).

### Linking mood and physiology

Although it is tempting to interpret associations between functional network organization and mood fluctuations in purely psychological terms, the present results demonstrate the contribution of physiological variables as well. Specifically, the participant’s fasting on approximately half of the scanning sessions had a significant effect on the expression of mood-related connectivity patterns. Although fasting did not influence mood fluctuations or connectivity–mood correlations, the effect of fasting on network expression serves as a reminder that subjective affect may be influenced by a variety of internal and external factors.

A related consideration is the multidimensional nature of subjective affect itself. Modern theories characterize affective experience along at least two distinct dimensions: valence (the ‘color’ of the experience) and arousal (the intensity of the experience; Feldman, 1995; Anderson *et al.*, 2003). Although the present analysis was designed to allow greater resolution with respect to mood by including all of the PANAS-X categories, it does not permit us to conclusively dissociate valence and arousal. Our results suggest an important role for arousal because PANAS-X categories related to ‘fatigue’ and ‘attentiveness’ loaded highly on the PLS-derived patterns (Figure 1B). This is consistent with the fact that the participant fasted prior to half of the scanning sessions. On fasting days, when the participants consumed neither food nor caffeine, he experienced lower arousal. Altogether, the present results point to a possible role in functional network organization as a method for dissociating neural representations of valence and arousal.

Interestingly, we found no other reliable associations between mood-related network patterns and exogenous lifestyle variables. For instance, network expression did not correlate with previous or same-evening gut health, stress or time spent outdoors. This is surprising given previous demonstrations that even short periods of time spent outdoors (e.g. nature walks) improve cognitive performance, such as working memory (Berman *et al.*, 2008). Lack of association with these variables may therefore suggest that subjective mood is not a simple reflection of environmental and physiological variables but must be considered from the perspective of neural signaling and network-wide interactions.

More generally, our findings open new questions about the physiological and psychological nature of subjective mood. Although we have demonstrated a link with hemodynamic correlations, mood depends on multiple physiological properties that are not accessible via fMRI, including electromagnetic rhythms and neurotransmitter signaling (Castrén, 2005). How proprioceptive and interoceptive signals are interpreted with respect to the external environment and in the context of emotional state remains an open question (Clark *et al.*, 2018). Our results suggest that distributed functional interactions provide a reliable signature of mood fluctuations, but how these functional interactions mediate the integration of sensorimotor and autonomic signals remains to be determined.

### Segregation and integration

Modern theories increasingly emphasize the role of system segregation in cognition and task performance (Shine and Poldrack, 2017; Wig, 2017). Resting-state system segregation, presumably reflecting autonomous processing in specialized circuits, is associated with greater cognitive ability, including episodic memory and fluid processing (Chan *et al.*, 2014; Gu *et al.*, 2015). Likewise, acquisition of automated skills is concomitant with increased segregation (Bassett *et al.*, 2015; Mohr *et al.*, 2016). Conversely, effective task performance, presumably requiring communication among distributed systems, is often associated with decreased segregation, including working memory (Vatansever *et al.*, 2015; Cohen and D’Esposito, 2016; Shine *et al.*, 2016a), cognitive control (Schultz and Cole, 2016; Shine *et al.*, 2016a), emotional/motivational processing (Kinnison *et al.*, 2012) and visuo-spatial attention (Spadone *et al.*, 2015). The present results suggest that a similar segregation–integration framework may apply to subjective affect. We find that daily differences in system segregation track subjective mood ratings, with positive mood associated with reduced segregation among intrinsic networks. While inter-network signaling may directly contribute to better mood, it may alternatively mediate mood via increased cognitive engagement.

### Methodological considerations

The present results are subject to several methodological considerations and potential limitations. Most importantly, these results are valid only for a single, 45-year-old male individual. Although the observed patterns are concordant with previous literature, further validation is needed in multiple individuals before we can be confident that the observed results will generalize to the rest of the population. We hope that the present report, much like several recent others, will prompt further investigation in single deeply phenotyped individuals (Laumann *et al.*, 2015; Poldrack *et al.*, 2015; Shine *et al.*, 2016b; Betzel *et al.*, 2017; Gordon *et al.*, 2017).

It is important to note that, because there are fewer observations (sessions) than variables (connections), relating connectivity patterns to mood scales is an underdetermined (ill-posed) problem. We have demonstrated the reliability of the results in several ways, including null non-parametric assessment of pattern stability and out-of-sample pairing, but further validation in independently collected data sets is necessary. With the advent of large imaging repositories, directly relating FC patterns to experimental manipulations is becoming an exciting new frontier in human brain mapping (Craddock *et al.*, 2015; Mišić and Sporns, 2016; Smith and Nichols, 2018). Statistical models, such as the one presented here, are still prone to overfitting and external replication is warranted.

## Conclusion

Altogether, our results demonstrate that functional network organization reflects daily fluctuations in subjective mood. Disruption of the intrinsic modular architecture, primarily driven by sgACC and components of the default mode network, is associated with positive mood. These results open exciting new questions about the confluence of proprioceptive and interoceptive signals, how they are mediated by functional neuroanatomy and how this system is disrupted in mood disorders.

## Materials and methods

### MyConnectome imaging data

The ‘MyConnectome Project’ is a deep phenotyping study of a single individual (male; right-handed; 45 years old) performed over the course of 1 year (Poldrack *et al.*, 2015). All data were downloaded from the project repository (https://myconnectome.org/wp/data-sharing/) on 13 April 2016. Brain imaging was performed at fixed times of the day (Tuesdays and Thursdays at 0730 h). Frames were censored (removed) if they had framewise displacement > 0.25 mm or if they were part of contiguous uncensored segments spanning <5 frames. Regressors included whole brain, white matter and ventricular signals and their derivatives, as well as 24 motion regressors derived by Volterra expression. To facilitate subsequent bandpass filtering (0.009–0.08 Hz), data were interpolated over the censored frames by least-squares spectral estimation (Poldrack *et al.*, 2015).

We analyzed the pre-processed, parceled fMRI time series for scan sessions 14–104. The pre-processing procedure is described in detail in (Laumann *et al.*, 2015; Poldrack *et al.*, 2015). The brain was parceled according to a custom partition optimized for the participant (Laumann *et al.*, 2015) using the procedure developed by Gordon *et al.* (2014) and Wig *et al.* (2014). The final parcellation consisted of 616 surface regions and 14 subcortical regions from Freesurfer’s subcortical parcellation, for a total of *N* = 630 regions of interest. Scan sessions were ~10 min long and the BOLD signal was sampled with a time to repetition (TR) of 1.16 s, yielding 518 time points per session. FC was defined as a zero-order linear Pearson correlation between regional BOLD time series.

### PANAS-X mood scales

Behavioral data, including self-rated affect, were collected on the same day as the imaging scans. Affective states were measured using the PANAS-X, a 60-item schedule completed using a 0–5 Likert scale (Watson and Clark, 1999). Following Watson and Clark (1999), we distilled the items into 2 general dimension scales (negative affect and positive affect) as well as 11 more specific scales (basic negative emotion scales: fear, hostility, guilt and sadness; basic positive emotion scales: joviality, self-assurance and attentiveness; other affective states: shyness, fatigue, serenity and surprise).

### Partial least squares

PLS analysis was used to relate FC patterns and mood patterns (Wold, 1966; McIntosh and Lobaugh, 2004; Krishnan *et al.*, 2011). The goal of the analysis is to identify weighted patterns of functional connections and mood scales that optimally covary with each other across scanning sessions. A form of reduced rank regression, PLS is closely related to canonical correlation analysis in the sense that both techniques aim to identify weighted mappings between two sets or blocks or variables (Worsley *et al.*, 1997; McIntosh and Mišić, 2013).

Connectivity and mood data were represented as two matrices X_*n* × *p*_ and Y_*n* × *q*_. Both matrices had *n* = 73 rows corresponding to the scanning sessions. The matrix X had *p* columns, corresponding to unique functional connections. Given *k* nodes, there were *P* = *k*(*k*-1)*/*2 connections. The matrix Y had *q* columns, corresponding to each of the PANAS-X scales. The matrices were standardized column-wise and a correlation matrix (XʹY) was computed. The matrix was then subjected to singular value decomposition (SVD; Eckart and Young, 1936)

(1) \begin{equation*} {\mathrm{X}}^{\prime}\mathrm{Y}=\mathrm{US}{\mathrm{V}}^{\prime } .\end{equation*}

The output of the decomposition are two orthonormal matrices of left and right singular vectors (U and V) and a diagonal matrix of singular values (S). The *i^th^* columns of U and V weigh the contribution of individual connections and behaviors, respectively. Collectively, the *i^th^* left and right singular vectors and singular value constitute a latent variable: a multivariate pattern that weighs the original variables such that they maximally covary with each other. The *i^th^* singular value is proportional to the total connectivity–behavior covariance accounted for by the latent variable. The effect size associated with a particular latent variable (*η*) can be estimated as ratio of the squared singular value (*σ*) to the sum of all squared singular values:

(2) \begin{equation*} {\eta}_i={\sigma_i}^2/\sum \limits_j{\sigma}_j^2 .\end{equation*}

#### Permutation tests

The statistical significance of the overall multivariate pattern was assessed by permutation tests (Nichols and Holmes, 2002). The rows of one of the data matrices (X) were randomly permuted, and a connectivity–mood correlation matrix was recomputed. The permuted correlation matrices were subjected to SVD as before, generating a singular value under the null hypothesis that there is no relation between FC and mood. This procedure was repeated 10 000 times to generate a null distribution of singular values. A *P*-value was estimated as the proportion of permuted singular values that surpass the original singular value.

#### Bootstrap resampling

Bootstrap resampling was used to estimate the reliability of individual connections and mood scale weights (Efron and Tibshirani, 1986; Milan and Whittaker, 1995). Sessions (i.e. the rows of X and Y) were sampled with replacement, and the resampled data matrices were used to generate correlation matrices that were then subjected to SVD as described above. The procedure was repeated 10 000 times, allowing us to construct a sampling distribution for each individual connection and behavior weight. To identify connections and mood measures that (a) made a large contribution to the overall pattern and (b) were stable across sessions, we calculated the ratio of a variable’s weight (*w_i_*) to its bootstrap-estimated standard error [SE(*w_i_*)], termed ‘bootstrap ratio’:

(3) \begin{equation*} {\mathrm{b}}_i={w}_i/\mathrm{SE}\left({w}_i\right) .\end{equation*}

If the bootstrap distribution is approximately Gaussian, the bootstrap ratio is equivalent to a *z*-score (Efron and Tibshirani, 1986). It is important to note that in the present analysis, where observations represent single-subject trials, the estimated sampling distributions yield CIs on fixed effects. As such, stability estimates may be vulnerable to natural or artefactual signal autocorrelation (e.g. due to movement or respiration).

#### Split-half resampling

To assess the generalizability of the estimated patterns, we performed split-half reliability testing (Kovacevic *et al.*, 2013). We randomly split sessions into two halves and calculated separate correlation matrices for each split (${\mathrm{X}}^{\prime }{\mathrm{Y}}_1$ and ${\mathrm{X}}^{\prime }{\mathrm{Y}}_2$). We then projected each matrix onto the original singular vectors U and V to estimate the corresponding singular vectors:

(4) \begin{equation*} {\displaystyle \begin{array}{l}{\mathrm{U}}_1={{\mathrm{X}}^{\prime }{\mathrm{Y}}^{\prime}}_1{\mathrm{V}\mathrm{S}}^{-1} \mathrm{and} {\mathrm{U}}_2={{\mathrm{X}}^{\prime }{\mathrm{Y}}^{\prime}}_2{\mathrm{V}\mathrm{S}}^{-1}\\ {}{\mathrm{V}}_1={\mathrm{X}}^{\prime }{\mathrm{Y}}_1{\mathrm{U}\mathrm{S}}^{-1} \mathrm{and} {\mathrm{V}}_2={\mathrm{X}}^{\prime }{\mathrm{Y}}_2{\mathrm{U}\mathrm{S}}^{-1}\end{array}}. \end{equation*}

Finally, we correlate the projected left and right split-half patterns (i.e. U_1_ and U_2_, and V_1_ and V_2_) across 1000 splits. The resulting correlations measure how reliably the FC and behavioral patterns can be paired with one another.

#### Cross-validation

As a final assessment, we estimated the out-of-sample correlation between connectivity and mood scores (Rahim *et al.*, 2017). The analysis was performed on 100 randomized train and test splits, with test sets representing 25% of the sample (19 sessions). Test data were projected on PLS-derived singular vectors, and the resulting test sample scores were correlated with each other. To assess the statistical significance of the correlation coefficient, we performed a permutation test with 1000 replications. Rows of the FC data matrix X were randomly permuted, and the procedure for estimating out-of-sample correlations between singular vectors was repeated. A *P*-value was estimated as the proportion of correlation coefficients generated for the randomly permuted samples that exceeded the original correlation coefficient.

### Modularity and segregation

Modularity (*Q*) is a commonly used quality function in community detection that quantifies the tendency for groups of nodes to form densely interconnected modules (Newman and Girvan, 2004):

(5) \begin{equation*} Q=\sum \limits_{ij}\left[{w}_{ij}-{p}_{ij}\right]\delta \left({c}_i,{c}_j\right) ,\end{equation*}

where *w_ij_* is the observed positive-valued connection weight between nodes *i* and *j*, while *p_ij_* is the expected connection weight between the nodes. The Kronecker delta function, *δ*(*c_i_, c_j_*), is equal to 1 when nodes *i* and *j* are assigned to the same community (*c_i_* = *c_j_*) and zero otherwise (*c_i_ c_j_*), ensuring that modularity is only computed for pairs of nodes belonging to the same community. The expected connection weight between pairs of nodes is defined according to the standard configuration model, such that

(6) \begin{equation*} {p}_{ij}=\frac{s_i{s}_j}{2m} ,\end{equation*}

where ${s}_i={\sum}_i{iw}_i$ is the strength of node *i* and $m={\sum}_{i,j>1}{w}_{ij}$ is total weight of all connections in the network. Under this null model, communities are considered to be of high quality if the constituent nodes are more highly correlated with each other than in a randomly rewired network with the same strength distribution and density.

An alternative measure of separation among putative communities is system segregation (Chan *et al.*, 2014). The measure (*S*) is defined as the normalized difference of mean within- and between-community connectivity:

(7) \begin{equation*} S={Z}_w-{Z}_b{Z}_w ,\end{equation*}

where *Z_w_* and *Z_b_* are the mean within- and between-community connection strengths, respectively.
